# *In vitro* methods for hazard assessment of industrial chemicals – opportunities and challenges

**DOI:** 10.3389/fphar.2015.00094

**Published:** 2015-05-05

**Authors:** Chin Lin Wong, Sussan Ghassabian, Maree T. Smith, Ai-Leen Lam

**Affiliations:** ^1^Centre for Integrated Preclinical Drug Development, The University of QueenslandSt Lucia, QLD, Australia; ^2^School of Pharmacy, The University of QueenslandWoolloongabba, QLD, Australia

**Keywords:** allergic contact dermatitis, epoxy resins, *in vitro* methods, skin sensitization, integrated hazard classification

## Abstract

Allergic contact dermatitis (ACD) is a delayed-type hypersensitivity immune reaction mediated by T-lymphocytes as a result of repeated exposure of an allergen primarily on skin. ACD accounts for up to 95% of occupational skin diseases, with epoxy resins implicated as one of the most common causes of ACD. Efficient high-throughput *in vitro* screening for accurate identification of compounds and materials that may pose hazardous risks in the workplace is crucial. At present, the murine local lymph node assay is the ‘method of choice’ for predicting the sensitizing potency of contact allergens. As the 3Rs principles of reduction, refinement, and replacement in animal testing has gained political and economic momentum, several *in vitro* screening methods have been developed for identifying potential contact allergens. To date, these latter methods have been utilized primarily to assess the skin sensitizing potential of the chemical components of cosmetic products with scant research attention as to the applicability of these methods to industrial chemicals, particularly epoxy resins. Herein we review the currently utilized *in vitro* methods and identify the knowledge gaps with regard to assessing the generalizability of *in vitro* screening methods for assessing the skin sensitizing potential of industrial chemicals.

## Introduction

Occupational skin diseases (OSDs) are a significant public health concern both in terms of employee pain and suffering as well as socioeconomic burden. In 2012 for the U.S. alone, the estimated annual direct and indirect costs of OSDs exceeded USD1 billion per annum (Lushniak, 2004; Cashman et al., 2012). Additionally, the cost of dermatological treatments is forecast to reach USD18.5 billion per annum by 2018 (Evers, 2013). These high socioeconomic costs have provided the impetus for development of efficient *in vitro* screening methods for accurately identifying chemicals with high skin sensitization risk so that their industrial use can be avoided, thereby reducing OSDs. One of the most commonly reported OSDs is contact dermatitis, which accounts for up to 95% of occupation-related skin diseases (Lushniak, 2000) in the areas of medicine, beauty products, manufacturing, and the construction industries (Gimenez-Arnau, 2011; Lowney and Bourke, 2011; Sosted, 2011).

Contact dermatitis is an inflammatory skin reaction resulting from direct contact with foreign substances, mainly affecting exposed skin areas such as the hands, arms, legs, and face (Belsito, 2005). Contact dermatitis can be classified into irritant contact dermatitis and allergic contact dermatitis (ACD). In this review, we address (i) ACD and its associated contact allergens, with particular attention on epoxy resins and their constituents and (ii) *in vitro* methods that may be used for risk assessment of ACD.

**FIGURE 1:**
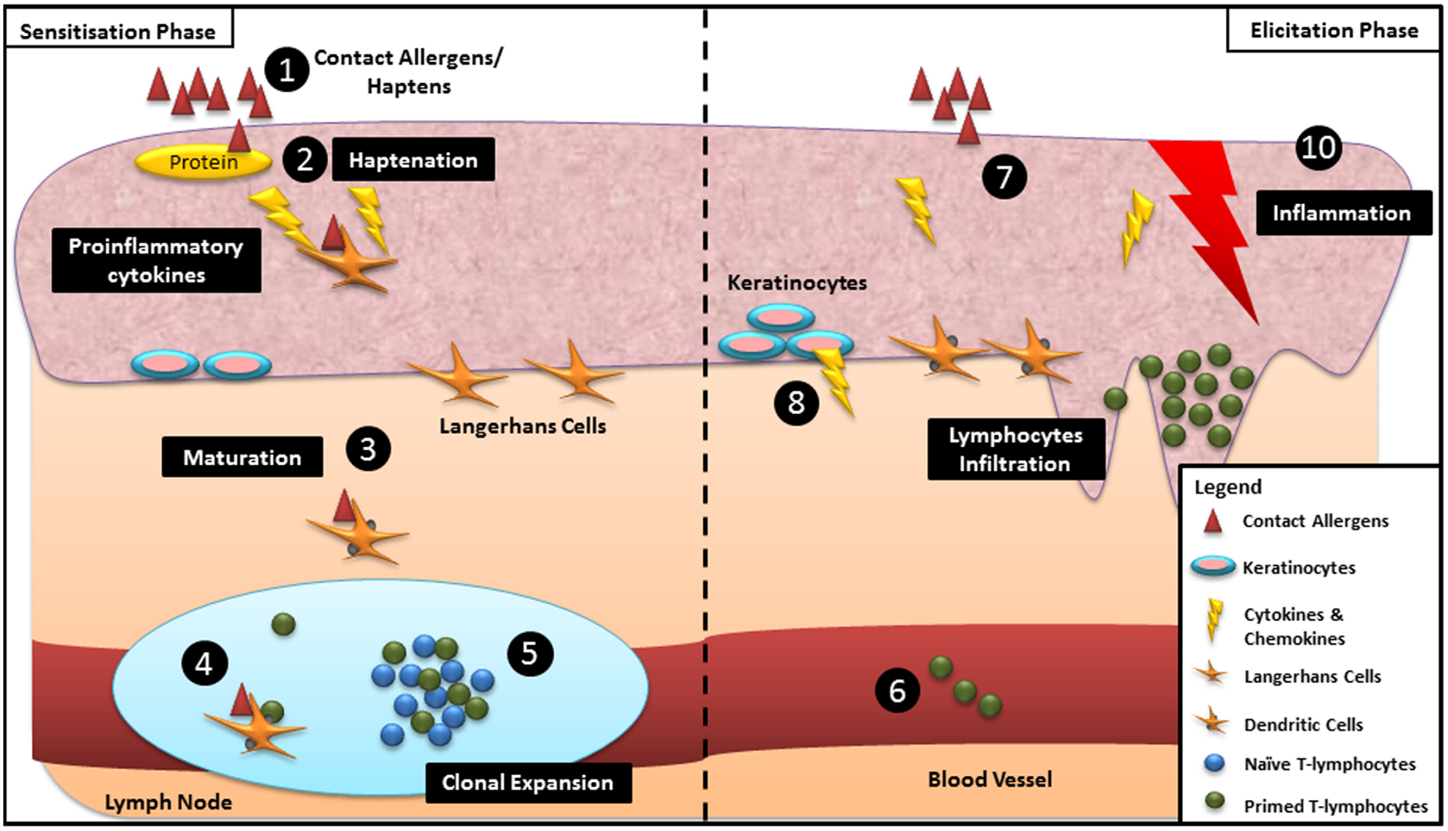
**Schematic overview of the mechanisms underpinning skin sensitization during sensitization and elicitation phases:** (1) Haptens gain access through the viable epidermis. (2) Binding of haptens and skin proteins. (3) Langerhans cells (LCs) bind to the hapten–protein complex and differentiate into matured dendritic cells (DCs) during migration to the lymph node. (4) LCs present haptenated protein to the naïve T-lymphocytes. (5) Clonal expansion of specific effector and memory T-cells. (6) Proliferated T-lymphocytes disseminate into the blood circulation resulting in sensitization of an individual. (7) Re-exposure of similar haptens to the same individual. (8) Release of proinflammatory cytokines and chemokines by epidermal cells. (9) Infiltration of T-cells from blood vessels into the site of contact. (10) Development of allergic contact dermatitis (ACD).

Allergic contact dermatitis is a type IV delayed hypersensitivity cutaneous immune reaction that is mediated by T-lymphocytes, and which occurs upon repeated skin exposure to contact allergens (Kimber et al., 2002a). Briefly, ACD develops in two stages, the sensitization phase and the elicitation phase (**Figure 1**; Toebak et al., 2009; Kimber et al., 2011). During the sensitization phase, contact allergens/haptens initially come into contact with the stratum corneum, the outermost layer of the skin and subsequently gain access to the body system through the viable epidermis. The invasion of haptens triggers the local release of proinflammatory molecules which subsequently induce the binding of haptens to skin proteins (Kimber et al., 2002a). The release of proinflammatory molecules also stimulates the disentanglement and subsequent migration of Langerhans cells (LCs) from the surrounding keratinocytes toward the hapten–protein complex (Schwarzenberger and Udey, 1996). The hapten–protein complex binds to the major histocompatibility complex (MHC) on LCs and is then transported into the lymph nodes via the afferent lymphatics (Toebak et al., 2009). During the transitory migration to the lymph nodes, the activated LCs differentiate into mature antigen presenting cells (APCs) resulting in morphological changes such as the loss of endocytic/phagocytic receptors and the upregulation of co-stimulatory molecules and MHC molecules (Toebak et al., 2009). The hapten–protein complex is presented by the APCs to the naïve hapten-responsive T-cells, followed by selective clonal expansion of effector and memory T-cells. The proliferated population of primed antigen-specific T-cells is then disseminated into the blood circulation resulting in the sensitization of an individual (Kimber et al., 2011). Elicitation is triggered when the haptens interact with either the same or a different skin site (Kimber et al., 2011). Upon re-exposure, epidermal cells release a cocktail of proinflammatory cytokines and chemokines which draw the hapten-specific T-cells from the peripheral circulation into the epidermal layer (Kimber et al., 2011). The infiltrating T-cells produce pro-inflammatory cytokines which in turn trigger the secretion of chemokines by keratinocytes, resulting in increased infiltration of T-cells from blood vessels into the epidermis leading to the development of ACD (Basketter and Maxwell, 2007; Toebak et al., 2009).

To date, more than 4000 chemical substances are linked to induction of ACD in humans (Cahill et al., 2012). The 18-year retrospective analysis of ACD patients identified a number of frequently defined contact allergens, some of which have been summarized in **Table 1** (Cahill et al., 2012). These chemicals have been compiled in several human patch tests series including the North American Series, the European Baseline Series, the International Standard Series and the Thin-layer Rapid Use Epicutaneous Tests (TRUE). In brief, these series identify chemical substances commonly implicated in the population of a given geographical area, to cause ACD (Spiewak, 2008).

**Table 1:** Common allergens and sources of exposure.

Allergens	Source
Epoxy resin system (ERS)	Adhesives, paints
Formaldehyde	Pesticides, home cleansers
Fragrance mix	Toiletries, cosmetics
Neomycin sulfate	Creams, deodorants
Nickel sulfate	Costume jewelry, tools


Epoxy resin-induced ACD was first reported in the 1950s, a time when there was extensive development of epoxy resin systems (ERSs) in industry (Broughton, 1965). In general, an ERS is comprised of an epoxy resin, hardener, reactive diluent, or other additives such as solvents, modifiers and fillers which together control the chemical and physical properties of the ERS (Geraut et al., 2009; Nixon et al., 2012).

Epoxy resin system components are the third most common allergen types for occupational ACD after chromates and rubber allergens, with ERS the primary source of ACD in the plastics manufacturing industry (Geraut et al., 2009). The prevalence of ERS-induced ACD by country is summarized in **Table 2**.

**Table 2:** Reported prevalence of occupational allergic contact dermatitis (ACD) due to epoxy resin systems (ERS).

Study period	Country	Study population (number of individuals)	Prevalence of ERS-induced ACD (%)	Reference
1993–2002	Australia	1354	3.0	Cahill et al. (2005)
1996–2006	North America	2540	0.9	Amado and Taylor (2008)
1997–2001	Norway	2336	1.0	Romyhr et al. (2006)
1999–2008	Portugal	2440	0.6	Canelas et al. (2010)
2001–2010	Denmark	219	8.2	Mose et al. (2012)
2001–2006	China	1354	8.5	Cheng et al. (2011)
2005–2009	Denmark	20 808	1.3	Bangsgaard et al. (2012)
2006–2008	Lithuania	816	1.5	Beliauskiene et al. (2011)

It was estimated that for individuals with ERS-associated ACD, ∼60–80% were sensitized to diglycidyl ether bisphenol A (DGEBA), an ERS that is widely used in industry (Björkner et al., 2011). This high prevalence resulted in the inclusion of DGEBA in the human patch test series since the 1960s (Geraut et al., 2009). Other epoxy resins including diglycidyl ether bisphenol F (DGEBF) and tetraglycidylmethylenedianiline, are also associated with induction of ACD (Geraut et al., 2009; Nixon et al., 2012).

Apart from epoxy resins, epoxy hardeners, predominantly polyamine compounds such as triethylenetetramine (TETA) and diethylenetriamine (DETA), as well as reactive epoxy diluents (e.g., phenyl glycidyl ether and *p*-tert-butylphenyl glycidyl; Geier et al., 2004), also cause ACD. A retrospective analysis of the records of 182 patients with ACD induced by epoxy resins over a 22-year period showed that 23.6% had developed an allergic response to epoxy hardeners (Jolanki et al., 2001). In a prospective study involving 92 individuals with suspected and/or prior exposure to ERS, patch tests showed that they were responsive to the epoxy diluents, 1,6-hexanediol diglycidyl ether (19.5%), and 1,4-butanediol diglycidyl ether (18.5%; Geier et al., 2004), highlighting cross-reactivity between epoxy compounds for induction of ACD in humans.

Although the high propensity of ERS to induce ACD is known, they are nevertheless used widely in commercial thermosetting products due to their strong adhesive bonding properties between different surfaces while exhibiting excellent resistance in harsh chemical and environmental conditions (Cahill et al., 2012). Worldwide demand for epoxy resins is forecast to reach ∼3 million tons by the end of 2017, with an estimated value of USD9.2 billion per annum (GIA forecasts the global market, 2012; Markets and Markets, 2014). The high global demand for epoxy resins is due to their ever increasing utility in a wide range of industrial applications including automotive coatings, electronic coatings, construction and adhesive products (Dietrich and Mirasol, 2012; GIA forecasts the global market, 2012). At present, research on assessment of the generalizability of *in vitro* tests developed for identifying the skin sensitizing potential of small molecules used in the toiletries and cosmetics industries, to that of epoxy resins and their components, is limited. Hence, this knowledge gap needs to be addressed. Herein, we review recent developments in non-animal tests for screening industrial chemicals for skin sensitization potential, with particular attention to the applicability of these tests to the epoxy resin chemical class.

## Contact Allergens Screening Approaches

Development of the first animal models more than 80 years ago, to substitute for human skin patch testing of chemical compounds as potential contact allergens, was a landmark in terms of minimizing if not altogether avoiding the need for human testing (Landsteiner and Jacobs, 1935). About 40 years later, animal models were introduced for assessing the sensitizing capacity of ERS (Thorgeirsson and Fregert, 1977; Thorgeirsson, 1978; Gamer et al., 2008; Ponten et al., 2009). At present, the murine local lymph node assay (LLNA) is the ‘gold standard’ for assessing the skin sensitization potential of contact allergens. However, the use of animals for this type of testing has provoked much ethical debate (Carlson et al., 2004; Basketter, 2009) and provided the impetus over the past decade for the development of *in vitro* methods to replace, reduce, and refine (3Rs) this type of animal testing (Flecknell, 2002). Although several of these non-animal testing methods are at the pre-validation stage, they have been used primarily to assess the skin sensitization potential of small molecules (molecular weights <500 Da) such as those used in the manufacture of cosmetic and toiletry products (Kaplan et al., 2012). However, their applicability for assessment of the skin sensitization potential of ERS is largely unexplored. Hence this knowledge gap needs to be addressed to enable the best method or combination of methods to be identified for the reliable assessment of the skin sensitization potential of epoxy resin compounds. In the following sections we review the current non-animal testing approaches that have been developed based upon key mechanistic events in the process of skin sensitization and address the limitations of these methods for assessing the skin sensitization potential of ERS.

### *In Chemico* Assays: Peptide–Chemical Interactions

Epoxy resins and/or epoxy resin composite materials, in common with other classes of haptens, react with skin proteins. The hapten–protein complex is then internalized and processed by LCs (Aleksic et al., 2007). Protein modification, in a process known as haptenation, is a key step in the initiation of skin sensitization (Chipinda et al., 2011). Majority of contact allergens are electrophilic in nature, consisting of Michael acceptors, S_N_Ar and S_N_2 electrophiles, Schiff base formers, or acylating agents, which underpin their ability to react with the nucleophilic amino acid residues of skin proteins (Chipinda et al., 2011; Lalko et al., 2012). For epoxy resins, the electrophilic epoxide groups react with the nucleophilic moieties of skin proteins via S_N_1 or S_N_2 type nucleophilic reactions (Obach and Kalgutkar, 2010).

**FIGURE 2:**
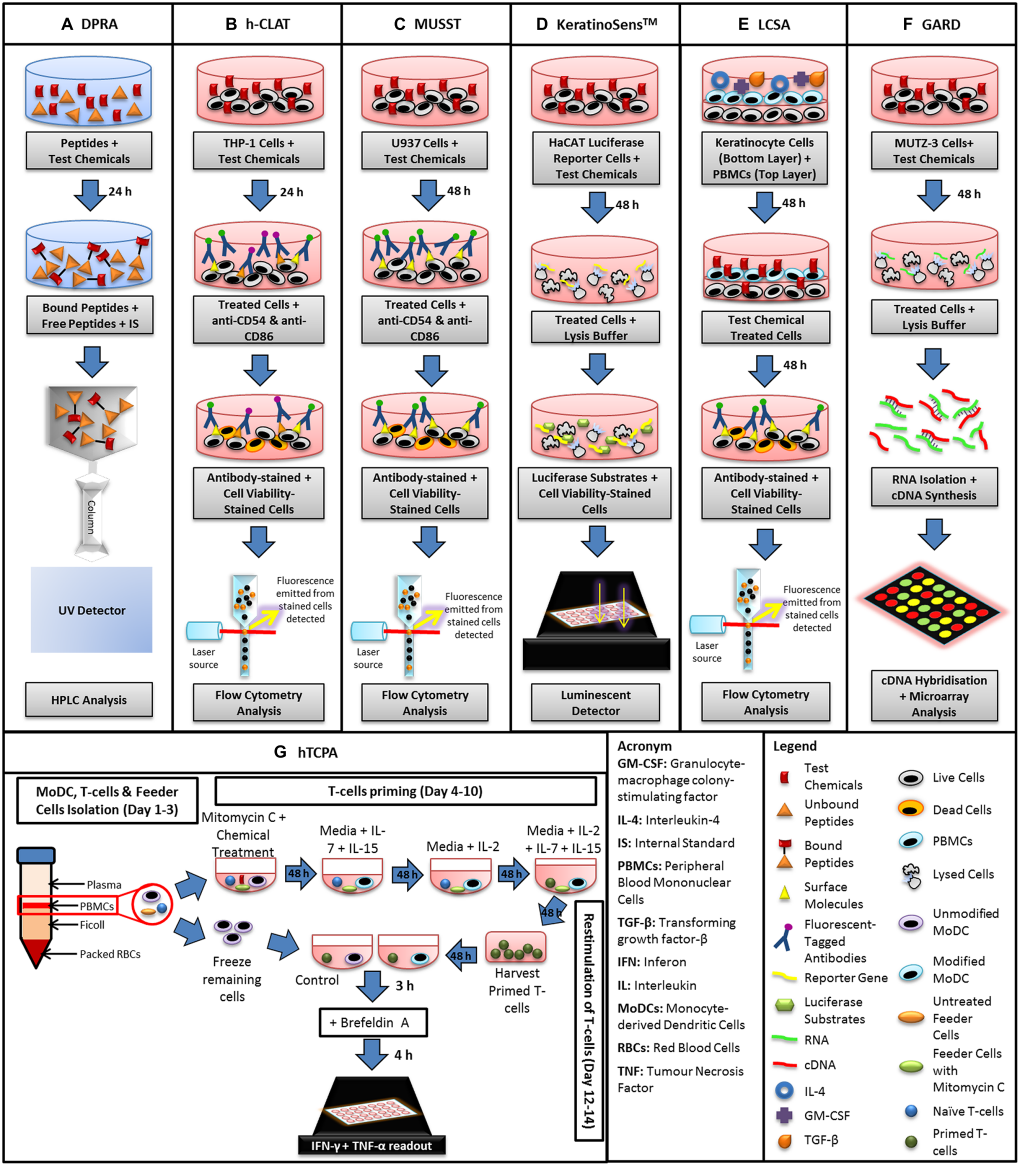
**Schematic diagram summarizing the steps involved in the conduct of *in vitro* assays currently available for assessment of skin sensitization potential. (A)** Direct peptide reactivity assay (DPRA), **(B)** human cell line activation test (h-CLAT), **(C)** myeloid U937 skin sensitization test (MUSST), **(D)** KeratinoSens^TM^, **(E)** loose-fit coculture-based sensitization assay (LCSA), **(F)** genomic allergen rapid detection (GARD), and **(G)** human T-cell priming assay (hTCPA).

This haptenation process is mimicked *in vitro* by the direct peptide reactivity assay (DPRA; **Figure 2A**) which assesses depletion of small proteins (peptides) secondary to their interaction with potential haptens (Gerberick et al., 2007). Briefly, in this model, synthetic peptides containing nucleophilic residues including cysteine or lysine are incubated with test chemicals at a pre-determined ratio for 24 h to allow the binding of the active side chain of the peptide to the hapten. Based upon the irreversible covalent bond formation that occurs between haptens and amino acid residues in proteins, the DPRA quantifies the amount of unbound (remaining) peptide in the reaction mixture using high performance liquid chromatography (HPLC). Subsequently, the quantification of the bound (depleted) peptides is determined as a measure of reactivity of the test chemical (Gerberick et al., 2004).

At present, the DPRA has been validated by the European Centre for the Validation of Alternative Methods (ECVAMs) for the assessment of contact allergens as a replacement for the *in vivo* LLNA (Troutman et al., 2011). A test guideline has been promulgated by the Organization for Economic Co-operation and Development (OECD) highlighting the generalizability of peptide reactivity with small molecules (OECD, 2015). However, the suitability of the DPRA test system for chemicals such as epoxy resins that contain an epoxide group remains to be assessed.

#### DPRA: Chemicals Tested to Date

Use of the DPRA to assess the ability of 82 compounds that are mainly used as ingredients in cosmetic and toiletry products, to deplete cysteine-, lysine-, and glutathione-based peptides, indicated a significant correlation between peptide depletion and their sensitizer potency as previously established from *in vivo* LLNA data (Gerberick et al., 2007).

Steps undertaken to improve the accuracy of the DPRA for identification of potential skin sensitizing chemicals have included incorporation of oxidizing agents such as horseradish peroxidase and hydrogen peroxide (HRP/P) as well as cytochrome P450 enzymes to metabolically activate unreactive haptens into their more reactive hapten form, a process that may take place in human skin *in vivo* (Bergström et al., 2007; Troutman et al., 2011). By incorporating HRP/P into the DPRA, 83% of 70 chemicals with known sensitizing potential were identified accurately as compared with the standard DPRA reported previously (89%; Troutman et al., 2011). This apparently reduced accuracy of the HRP/P-added DPRA analysis is misleading, however, as the initial chemical set used to evaluate the previous DPRA prediction model did not include pre-/pro-haptens (Gerberick et al., 2007).

More recent refinements aimed at increasing the robustness of the DPRA to identify skin sensitizing chemicals include using pH conditions that more closely mimic human skin pH and measurement of concomitant chemical-specific mass changes indicative of peptide adduct formation (Dietz et al., 2013). In other work, the rate constant for reactivity of various test chemicals with the DPRA peptide was determined to assess whether quantitative kinetic reactivity data generated by measuring cysteine depletion at multiple test chemical concentrations and at various incubation times, were correlated with their potency as sensitizers (Roberts and Natsch, 2009; Natsch et al., 2014). However, drawbacks of this approach are that chemical reactivity varies markedly between various functional groups and the reaction rate of test chemicals with the DPRA peptide may not be linearly related to their *in vivo* sensitization potency (Roberts and Natsch, 2009).

#### DPRA: Application to ERS

While cysteine and lysine are the most widely utilized peptides for the *in vitro* DPRA, other modified peptides have been investigated. More recently, the utility of the DPRA for classifying the sensitizing capacity of several epoxies including novel analogs of DGEBF and phenyl glycidyl ether (PGE), has been examined using a synthetic peptide, viz PHCKRM (Pro-His-Cys-Lys-Arg-Met). The extent of peptide (PHCKRM) depletion by six novel epoxy analogs and the parent epoxide, PGE, was correlated with the sensitizing potency of these epoxies determined using *in vivo* LLNA assessment (Niklasson et al., 2009). The strong sensitizer, PGE produced 88% peptide depletion whereas the weak epoxide sensitizers, butyl glycidyl ether, and butenyl glycidyl ether produced 46 and 54% peptide depletion, respectively (Niklasson et al., 2009). In a DPRA evaluation of DGEBF (containing two epoxide groups) and two variants (Variant A and Variant B) using the same synthetic peptide (PHCKRM), the thiol (cysteine) binding of DGEBF and its variants appeared to be affected by the terminal epoxide groups (O’Boyle et al., 2012). Variant A (DGEBF without terminal epoxide groups) did not react with free thiols whereas variant B (DGEBF with one terminal epoxide group) did react with thiol groups albeit to a slightly lesser extent than the diepoxide DGEBF. Interestingly, the reaction rate for DGEBF that contains two terminal epoxide groups was slightly faster than that of variant B. These findings are aligned with the sensitizing capacity of DGEBF and its variants determined using the LLNA and the KeratinoSens^TM^ assay (O’Boyle et al., 2012).

To date, reports on the applicability of the incorporation of enzymes into the DPRA, as a means of bioactivation for assessing the skin sensitization potential of epoxy resins, are lacking. It is known that the enzyme, epoxide hydrolase, catalyzes the hydrolysis of epoxides to their respective dihydrodiol metabolites which react readily with skin proteins. Conversely, the enzyme, glutathione-*S*-transferase catalyzes the detoxification of epoxides by formation of glutathione conjugates (Obach and Kalgutkar, 2010). Hence, future investigation involving incorporation of epoxide hydrolase and/or glutathione-*S*-transferase into the DPRA for analysis of epoxy resin compounds is warranted, to more closely mimic possible bioactivation and deactivation processes within human skin that produce reactive electrophilic intermediates and detoxified species, respectively.

Issues relating to the poor aqueous solubility of industrial compounds that have high octanol/water partition coefficients, present another obstacle for use of DPRA to assess compounds such as epoxy resins. Although various solvents including dimethylsulfoxide (DMSO), methanol and acetonitrile have been used to dissolve lipophilic compounds, only small volumes of these solutions can be used due to their limited miscibility with an aqueous solution of the peptide to be depleted. To that extent, microemulsion systems have potential to improve miscibility between an organic solution of a lipophilic test compound and that of an aqueous peptide solution; preliminary data suggest that this approach is worthy of further investigation (Merckel et al., 2010).

Unacceptable modulation of the test systems by organic solvents limits the range of solvents that can be used for dissolution of epoxy resins. For example, organic solvents routinely used in laboratories inhibit cytochrome P450-mediated metabolic reactions, and may potentially fail to activate the enzyme-dependent sensitizing chemicals in the test system (Li et al., 2010; Troutman et al., 2011). DMSO is unsuitable for use in the DPRA as its high reactivity means that it may react with assay peptides resulting in false positive results. The use of DMSO in the DPRA would require an additional costly step of purging the reaction system with an inert gas such as argon, to prevent oxidation of DMSO (Niklasson et al., 2009).

### *In Vitro* Assays: Cell-Based Models

Human LCs and dendritic cells (DCs) play key roles in skin sensitization (Coutant et al., 1999). Hence, there has been considerable research attention on development of *in vitro* systems that mimic the roles of LCs and DCs in skin sensitization. Initial *in vitro* assays using LCs/DCs were limited due to the scarcity of available LCs and inter-donor variability of DCs (Yoshida et al., 2003). These factors were compounded by between-laboratory variability in cell isolation and cell culture techniques, which led to assay reproducibility problems (Yoshida et al., 2003). The inter-donor variability was circumvented by the use of human myeloid cell lines, such as KG-1, THP-1, MUTZ-3, and U937 that have the ability to differentiate into cells with DC-like characteristics (Hu et al., 1996; Koss et al., 1996; Yoshida et al., 2003). Several *in vitro* model systems using human cell lines to assess the skin sensitizing potential of contact allergens have been developed. These include the human cell line activation assay (h-CLAT), myeloid U937 skin sensitization test (MUSST), the KeratinoSens^TM^ test (**Figures 2B–D**) and the LuSens which were under ECVAM evaluation (Ade et al., 2006; Ashikaga et al., 2006; Sakaguchi et al., 2006; Python et al., 2007; Emter et al., 2010; Bauch et al., 2012). These methods have been reviewed extensively by others (Mehling et al., 2012; Vocanson et al., 2013), and hence will not be covered in this review.

#### Loose-Fit Coculture-Based Sensitization Assay (LCSA)

An allergen-sensitive *in vitro* method that combines two layers of cells, termed the loose-fit coculture-based sensitization assay (LCSA), was developed using human primary keratinocytes from healthy donors, and mobile DC-like cells viz peripheral blood mononuclear cells (PBMCs; **Figure 2E**; Schreiner et al., 2008). As keratinocytes are proposed to have a role in haptenation via maturation of DCs, this assay has the advantage of being able to detect prohaptens such as isoeugenol (Schreiner et al., 2008), that are not detected by many *in vitro* model systems. In short, inclusion of keratinocytes in this two-tiered cell-based system facilitated metabolic activation of prohaptens into sensitizing agents akin to that which occurs in the skin *in vivo* (Wanner et al., 2010).

Similarly to MUSST and h-CLAT (as depicted in **Figures 2B,C**), LCSA quantifies the increase in expression of the cell surface marker, CD86 (Schreiner et al., 2007). Additionally, LCSA accuracy and sensitivity for assessing metal allergens such as nickel and cobalt, was improved by measuring accumulation of the proinflammatory cytokine, interleukin-6 (IL-6) and the chemokine macrophage inflammatory protein 1-β (MIP-1β; Schreiner et al., 2008). In a comparative evaluation of the *in vitro* LCSA relative to the *in vivo* LLNA for assessing the skin sensitizing potential of a group of textile disperse dyes, both methods identified 87.5% of these dyes as having skin sensitizing potential. Hence, the LCSA is a promising *in vitro* method for identifying agents with skin sensitizing potential for use in combination with other non-animal testing methods (Sonnenburg et al., 2012). However, the current challenges in using the LCSA include the necessity to obtain keratinocytes and PBMCs from healthy human donors which makes the method susceptible to inter-donor variability. Additionally, the complexity and time required for seeding keratinocytes and PBMCs in this co-culture assay makes it low throughput and so future innovation is required to adapt the LCSA to high throughput format.

#### Genomic Allergen Rapid Detection (GARD)

Apart from quantification of changes in cell surface expression of molecules of interest, genomic methods may offer an alternative or complementary *in vitro* testing paradigm. For example, genomic allergen rapid detection (GARD) employs the myeloid cell line, MUTZ-3 that resembles skin DCs with respect to transcriptional profiles and the ability to activate specific T-cell populations (**Figure 2F**; Johansson et al., 2013). GARD uses a complete genome expression array approach to measure expression levels of 200 transcripts involved in the activation of various signaling pathways involved in skin sensitization.

Unlike the KeratinoSens^TM^, MUSST and h-CLAT *in vitro* methods that use specific markers for classifying sensitizers, GARD utilizes ‘biomarker signatures’ for identifying skin sensitizers, thereby potentially increasing the predictive ability of the method. An added advantage of GARD is that it can distinguish respiratory and skin allergens by their unique biomarker signatures (Johansson et al., 2013). Encouragingly, use of GARD to assess 38 chemicals with known skin sensitization potential in a preliminary study, showed that the accuracy, sensitivity, and specificity of the method was high at 99% (Johansson et al., 2011).

Recently, Albrekt et al. (2014) stressed that chemical reactivity properties were key factors for consideration when developing *in vitro* screening models of chemical sensitizers. Sensitizing chemicals were divided into groups based upon their mechanistic reactivity and assessed against various cell-signaling pathways using the GARD assay. Interestingly, different chemical reactivity groups induced differential changes in various cell signaling pathways, particularly those involved in cell cycling and metabolism. Potency in modulating these pathways appeared to be correlated with skin sensitization potential (Albrekt et al., 2014). However, care is required to avoid over-interpretation of these associations with respect to potential sensitizer classification. More work is clearly required using larger numbers of chemicals with a broad range of functional groups of varying reactivity, as well as a range of concentrations and reaction times. Nevertheless, the GARD assay can provide invaluable information on the various cell signaling pathways underpinning the sensitization process which is invaluable in informing further development of *in vitro* sensitization test methods. Future research is warranted to assess the extent to which the epoxide group in ERS will modulate cell-signaling responses based upon their reactivity domain and/or their sensitizing potency.

#### T-cell Activation Model

During skin sensitization, specific effector and memory T-cells are activated by DCs triggered by sensitizing agents. While activation and proliferation of T-cells reflect the ultimate step in inducing sensitization, there are very few assays that address this aspect of the sensitization process. At present, only the *in vivo* LLNA is used widely to evaluate the activation and expansion of T-cells. More recently, an *in vitro* assay known as the human T-cell priming assay (hTCPA) was developed to assess T-cell responses initiated by contact allergens (**Figure 2G**; Dietz et al., 2010; Richter et al., 2013). The hTCPA uses naïve T-cells isolated from PBMCs of healthy donors that are depleted in CD25^+^ and CD45RO^+,^ a T-cell population responsible for regulating hapten-specific interferon-γ (IFN-γ)-producing T-cells in lymph nodes (Vocanson et al., 2013). The modified T-cells are co-cultured with hapten-treated monocyte-derived DCs at two stages, priming and re-stimulation. After re-stimulation, the increase in T-cell production and the cytokines, IFN-γ and TNF-α (tumor necrosis factor-α), are quantified using an enzyme-linked immunosorbent assay (ELISA) and an intracellular cytokine assay (Richter et al., 2013; Vocanson et al., 2013).

The hTCPA has been used successfully to assess the skin sensitizing potential of the strong sensitizers, 2,4-dinitrochlorobenzene (DNCB), 2,4-dinitrobenzenesulfonic acid (DNBS) 2,4,6-trinitrobezene sulfonic acid (TNBS), and moderate/weak sensitizers, fluorescein isothiocyanate (FITC), and α-hexyl cinnamaldehyde (HCA) as well as the non-sensitizers, methyl salicylate, DMSO, and sodium lauryl sulfate (SLS; Vocanson et al., 2014). Hence, the hTCPA has potential as an *in vitro* method for assessing the sensitizing potential of contact allergens. However, similar to the LSCA, this method is time-consuming and fraught with difficulty in assay reproducibility due to the scarcity of T-cell donors and inter-donor variability. More work is warranted to assess the applicability and generalizability of this cell-based model system using a larger number and a wider range of chemical compound classes. For example, the hydrophobic compound, DNCB that reduced DCs uptake did not stimulate T-cell proliferation (Dietz et al., 2010). While the use of nanoparticle encapsulation of lipophilic compounds significantly increased the ability of DNCB to stimulate T-cell proliferation and thus increase the assay sensitivity (Vocanson et al., 2013), inclusion of this additional step adds another level of complexity and increases the cost of the assay.

#### Cell-Based Models and ERS

Despite significant progress in the development and optimization of non-animal testing assays, a major limitation in their use for accurately identifying the skin sensitizing capacity of test compounds, is poor water solubility, particularly for aqueous cell-based assays (McKim et al., 2012). To date, few ERS compounds have been assessed using cell-based *in vitro* model systems. While the KeratinoSens^TM^ assay has been used successfully to classify the skin sensitizing potential of DGEBA, DGEBF, and PGE (Delaine et al., 2011; O’Boyle et al., 2012; Natsch et al., 2013) to match the LLNA results, the generalizability of other *in vitro* cell-based methods reviewed herein is a knowledge gap and remains to be determined.

Maintaining a suitable balance between the final solvent composition, test compound solubility and deleterious solvent-related effects within the assay, is pivotal for generating meaningful data on skin sensitization potential. In general, the solvent-related issues associated with *in vitro* assays relate to toxicity and/or solvent-mediated modulation of the assay response, thereby confounding assay readouts resulting in inaccurate assessment of skin sensitization potential. High solvent concentrations in cell-based assays adversely affect cellular integrity, resulting in cell death (Tapani et al., 1996; Galvao et al., 2014). Concentration-related toxic effects of the solvent need be evaluated to identify the maximum ‘no effect’ levels for each *in vitro* assay. The balance between acceptable solvent percentage in the aqueous cell-based test system whilst maintaining solubility of high molecular weight and low solubility test compounds, particularly industrial epoxy resins is yet to be adequately addressed. This issue is arguably the most significant obstacle to be overcome in adapting current *in vitro* skin sensitization assays to assessment of epoxy resin hazard risk.

## Skin Models

While selection of solvents compatible with *in chemico* assays may improve the ability of the DPRA to identify epoxy resins that have skin sensitizing properties, it is more difficult to attain a suitable balance between epoxy resin solubility and cell viability in aqueous culture-based assays. Moreover, future investigation is required regarding the fact that most test compounds are applied in solution to *in vitro* assays which may not necessarily be reflective of the situation in humans where there may be topical application of the compound in the solid state to the skin. To address this issue, the reconstructed human epidermis (RHE) has considerable potential. The RHE comprises an acellular dermal matrix mimicking the human skin epidermis layer. It has been used together with cytokines and growth factors to better represent the human skin micro-environment (Gibbs et al., 2007). Preliminary data using the RHE system showed that it was responsive to known sensitizers (Uchino et al., 2011).

More recently, EpiSensA, an *in vitro* skin sensitization assay that utilizes a commercially available RHE has become available (Saito et al., 2013). In brief, using this skin model system, skin sensitizing potential of test compounds is assessed based upon changes in the expression of genes related to the cellular stress response. Preliminary data from 16 test compounds were promising (Saito et al., 2013). Despite considerable progress, the challenge remains for a more complete human skin model system to become available that has a high degree of accuracy for correctly identifying and classifying the skin sensitization potential of novel compounds. This challenge is multi-factorial encompassing inter-individual differences at both the cellular and molecular levels such as genotypic variation, differences in epidermal thickness and metabolic activity of the skin, as well as inter-individual differences in rates of skin cell differentiation (Gibbs et al., 2007). Nevertheless, EpiSensA has promise for improving *in vitro* assessment of the skin sensitizing properties of compounds with poor aqueous solubility such as epoxy resins.

## Challenges in Assessing Epoxy Resin Compounds Using non-Animal Testing Systems

Apart from use of RHE model systems, the accuracy of *in vitro* methods for skin sensitization assessment of industrial chemicals may be improved by including multiple assay readouts using an ‘assay panel’ approach (Natsch et al., 2009; Jaworska et al., 2011; Bauch et al., 2012). However, questions on the generalizability of these *in vitro* methods to accurately identify chemicals containing very different functional groups, is as yet unclear. In particular, most *in vitro* methods were developed and evaluated using small molecule chemicals that are widely utilized in the manufacture of cosmetic and toiletry products. This is a significant limitation as it has now been shown that different functional groups with varying chemical reactivity produce differential engagement of cell signaling pathways (Albrekt et al., 2014).

For example, a dataset of 145 chemical compounds assessed using the KeratinoSens^TM^ and MUSST assays, those that were preferentially lysine-reactive resulted in false negatives (Natsch et al., 2013). These findings mirror work by others (Migdal et al., 2013) whereby chemicals with high reactivity toward cysteine, and not lysine, activated the nuclear factor erythroid-derived 2-related factor 2 (Nrf2)-ARE pathway in THP-1 cells, a well-known toxicity pathway activated by skin sensitizers (Natsch, 2010) that underpins the design principles of both the KeratinoSens^TM^ and LuSens tests. ERS compounds such as DGEBA, DGEBF, and PGE react selectively with thiol groups (cysteine; O’Boyle et al., 2012; Natsch et al., 2013). Hence, the KeratinoSens^TM^ and LuSens assays that are based on the aforementioned pathway are worthy of future investigation for their applicability and reliability to assessment of the skin sensitizing potential of epoxy resins.

However, it is important to bear in mind that a single stand-alone method based upon a single mechanistic pathway to assess novel derivatives of ERS compounds is fraught as the novel derivatives may produce skin sensitization by a different mechanistic pathway. To address this issue, ECVAM recommendations are that the KeratinoSens^TM^ be used as part of an integrated assessment approach that may also include the DPRA (ECVAM, 2014). Hence, future research is required to assess the applicability of current *in vitro* methods to assess the skin sensitizing potential of a broader range of chemical compounds as a means to identify the most appropriate *in vitro* assays and assay readout ranges, for establishing benchmarks to use for classifying the skin sensitization potency of novel compound classes.

Another consideration to this discussion is the inherent accuracy of the LLNA itself with respect to existing human data. The LLNA is widely utilized as the benchmark for evaluating the predictive accuracy of non-animal methods. However, when compared against the human maximization and patch test, the accuracy of the LLNA was 72% (Anderson et al., 2011). More recently, a retrospective comparison of a moderately large dataset (>100) of test compounds revealed an 82% predictive accuracy for LLNA when compared with established human data (Urbisch et al., 2014). In other work, use of an integrated testing strategy-based on data from ‘2 out of 3 *in vitro* prediction models’ resulted in a higher overall accuracy (≥90%) when compared with human data, as opposed to ≤83% using the LLNA dataset (Bauch et al., 2012; Urbisch et al., 2014). Factors potentially contributing to the discordance between human and LLNA data include the difference in skin penetration rates between the mouse and human, as well as the application method of the test compounds on the skin (Anderson et al., 2011; Delaine et al., 2011). The volatility and cytotoxicity of compounds such as the components of ERS, could affect potency outcomes given the open nature of substance application to the mouse ear in the LLNA in contrast with the occluded dressing used in human patch tests (Delaine et al., 2011). Hence, where possible, it is important to compare data produced by various *in vitro* skin sensitization tests with human data where available rather than relying solely on comparisons with LLNA data.

## Integrating Non-Animal Assay Readouts: Classifying Potential Skin Sensitizers

The OECD has proposed that the hazard classifying system for chemicals should consider the potential severity of allergic manifestations from human and animal-based epidemiological data [Organisation for Economic Co-operation and Development (OECD), 1998]. A strong sensitizer is defined as a compound that has a high occurrence of sensitization within an exposed population whereas low to moderate sensitizers produce a low or moderate frequency or severity of sensitization [Organisation for Economic Co-operation and Development (OECD), 1998]. At present, this chemical classification system is based solely on the ‘gold standard’ LLNA which assesses the potency of skin sensitizers based on the extent to which they induce T-cell proliferation in the auricular lymph nodes of mice (Kimber et al., 2002b). Current and future research aimed at gaining a deeper understanding of the various cellular and immunological mechanisms and their interplay that contribute to the extent of sensitization evoked, is essential. Such new knowledge will be invaluable for informing future research aimed at optimization of *in vitro* methods for hazard identification of industrial chemicals, particularly ERS, as well as enable quantitative risk assessments to be performed (Kimber et al., 2011).

**FIGURE 3:**
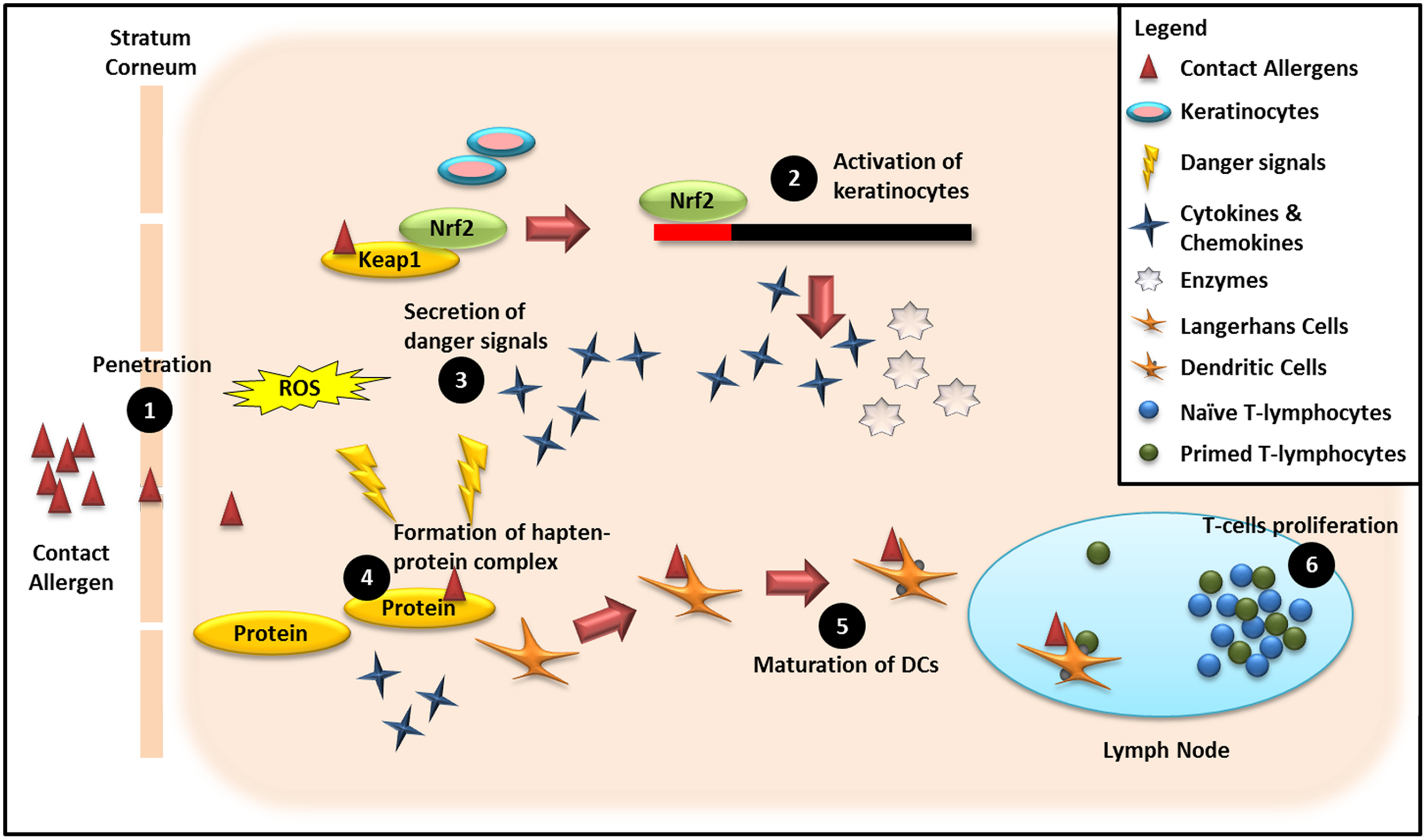
**Schematic overview of mechanisms underpinning non-animal methods for assessing the sensitizing potential of chemical compounds.** (1) Penetration of haptens through the viable epidermis: Quantitative Structure–Activity Relationship (QSAR). (2) Activation of keratinocytes in the epidermal layer by haptens: KeratinoSens^TM^, LuSens. (3) Secretion of danger signals from the epidermal compartment due to invasion of haptens: ROS production, genomic fingerprints, and proteomics biomarkers. (4) Formation of the hapten–protein complex: DPRA, peroxidase peptide reactivity assay (PPRA) and QSAR, Allergen-peptide/protein interaction assay (APIA). (5) Maturation of DCs when migrating from epidermal compartment to auricular lymph nodes via afferent lymphatics: h-CLAT, MUSST, LCSA. (6) T-cell proliferation in auricular lymph nodes: hTCPA.

The available non-animal testing methods for assessing the various stages of ACD are summarized in **Figure 3**. This schematic diagram clearly shows that single testing methods are unable to evaluate potential cross-talk between the various phases of the skin sensitization process. Thus, a single *in vitro* test representative of a single event in the human skin response to contact allergens cannot adequately capture the complexity of the human response to a contact allergen, thereby potentially leading to generation of false negative results (Aeby et al., 2010). Thus, a panel of complementary non-animal tests that together mimic the complexity inherent in *in vivo* test methods (e.g., LLNA, human patch test), has considerable potential utility as a screening tool for more accurately classifying novel compounds as extreme, strong, moderate or weak sensitizers.

An integrated hazard classification scheme involving assessment of multiple steps in the skin sensitization process, including bioavailability, structural alerts, formation of hapten–protein conjugates, DC maturation, and T-cell proliferation, has been proposed (Kimber et al., 2003; Jowsey et al., 2006). Using this approach, greater weight is given to *in vitro* tests that produce quantitative data. An index of sensitizing potency is calculated based upon the product of values obtained from each test representing a key step in the skin sensitization process, for comparison of skin sensitization potency with the corresponding mouse LLNA data (Kimber et al., 2003; Jowsey et al., 2006). Various non-animal test combinations proposed for identifying potential skin sensitizers are summarized in **Table 3**.

**Table 3:** *In vitro* methods used in combination for classifying and predicting skin sensitization potential of novel chemical compounds.

Combination methods	Description	Accuracy	Reference
(a) Peptide reactivity (b) Cell-based ARE^†^ assay (c) TIMES-SS^‡^ computer modelling (d) Calculated octanol-water partition coefficient	• Scores of 0–4 for each individual test • A binary system is applied for *in silico* test results	88% (based on LLNA data) (116 test substances)	Natsch et al. (2009)

(a) DPRA (b) LuSens (similar principle with KeratinoSens^TM^ assay) or KerotinoSens^TM^ assays (c) h-CLAT or MUSST	• A sensitizer if DPRA and LuSens yield negative results and MUSST is positive • If contradictory results between DPRA and LuSens, or h-CLAT, then weight of evidence approach is used	94% (based on human data) 83% (based on LLNA data) (54 test substances)	Bauch et al. (2012)

Bayesian network Integrated Testing Strategy (a) TIMES^§^ (b) DPRA (c) ARE luciferase activity (d) MUSST	• Adaptive testing strategy where the choice and sequence of tests performed are based on available information • Reduces uncertainty of the sensitizing capacity of a test substance before proceeding to the experiment.	–	Jaworska et al. (2011)

^†^ARE, antioxidant *response element.*

^‡^TIMES-SS, tissue metabolism simulator for skin sensitization.

^§^ TIMES, tissue metabolism simulator.

## Conclusion

A strategy encompassing the integration of readouts from multiple *in vitro* tests as a means to improve the accuracy for identification of novel compounds that are contact allergens has merit. However, implementation of such a strategy requires extensive validation and assessment of its generalizability for multiple chemical classes before gaining widespread acceptance. Additionally, use of an integrated panel of *in vitro* methods to screen large numbers of industrial chemicals is likely to be unattractive from a cost and time perspective and so development of a hierarchy of individual high throughput *in vitro* tests is needed.

At present, single *in vitro* assays in high throughput format enable large numbers of compounds to be screened in a short time frame. However, the choice of *in vitro* method for screening purposes, either as part of an integrated or hierarchical strategy, should be informed by knowledge of the chemical class/domain. In conclusion, the choice of *in vitro* methods for inclusion in a panel for assessing skin sensitization potential will be the best balance between predictive power of the selected tests relative to the time and cost of generating the data and its value to the organization that requires the data.

## Author Contributions

CW and AL wrote the manuscript. All authors reviewed and commented on the manuscript drafts.

## Conflict of Interest Statement

The authors declare that the research was conducted in the absence of any commercial or financial relationships that could be construed as a potential conflict of interest.
